# A Case Report of Congenital Insensitivity to Pain and Anhidrosis (CIPA)

**Published:** 2012

**Authors:** Mehran KARIMI, Razieh FA LLAH

**Affiliations:** 1 Associate Professor of Pediatrics, Department of Pediatrics, Shahid Sadoughi University of Medical Sciences, Yazd, Iran; 2 Associate Professor of Pediatric Neurology, Department of Pediatrics, Shahid Sadoughi University of Medical Sciences, Yazd, Iran

**Keywords:** HSAN type IV, Congenital insensitivity to pain, Anhidrosis

## Abstract

Congenital insensitivity to pain and anhidrosis (CIPA) or hereditary sensory autonomic neuropathies type IV (HSAN type IV) is an extremely rare autosomal recessive disorder initially described by Swanson in 1963.

We report a 2.5-year-old boy with clinical features of CIPA as the first case in Iran. The symptoms included recurrent episodes of hyperthermia and unexplained fever that began in early infancy, anhidrosis (inability to sweat), profound loss of pain sensitivity, neurodevelopmental delay, unconscious self-mutilation of fingers, lips and tongue, corneal lacerations, palmar hyperkeratosis, non-painful fracture and joint deformities in the right ankle. Tearing, deep tendon reflexes and motor and sensory nerve action potentials were normal.

Prenatal screening is the sole accessible option to prevent the birth of an affected child as no cure is available. Early recognition of CIPA patients and its orthopedic complications, prevention of accidental injuries, regular visual and eye follow-up and specific dental management could be useful in the reduction of frequency and severity of complications.

## Introduction

Hereditary sensory autonomic neuropathies (HSAN) occur much less frequently than hereditary motor sensory neuropathies. They have been categorized into five types based on inheritance pattern, time of presentation, clinical and electrophysiological features, metabolic defects and specific genetic markers (1).

Congenital insensitivity to pain and anhidrosis (CIPA) or HSAN type IV is an extremely rare autosomal recessive disorder initially described by Swanson in 1963 (2). The incidence of this disorder has been estimated to be 1 in 25, 000 population (3).

CIPA is also known as familial dysautonomia type II which is secondary to a mutation in the neurotrophic tyrosine kinase receptor type 1 (NTRK1) gene, located in chromosome 1 which encodes one of the receptors for the nerve growth factor.

The diagnosis of CIPA is based on the clinical presentation, pharmacological test (intradermic reaction to 1:10, 000 histamine) and neuropathological exam in electron microscopy (absence of unmyelinated fibers, reduction in the number of small myelinated fibers, and normal distribution of large myelinated fibers) and detecting of mutations on the NTRK1 gene represents as the last diagnostic step (1,4, 5).

Three cases of HSAN type II were reported in Iran previously (6, 7) and here we report a case of HSAN type IV as the first case of this type of disorder in Iran. 

## Case presentation

**Fig1:**
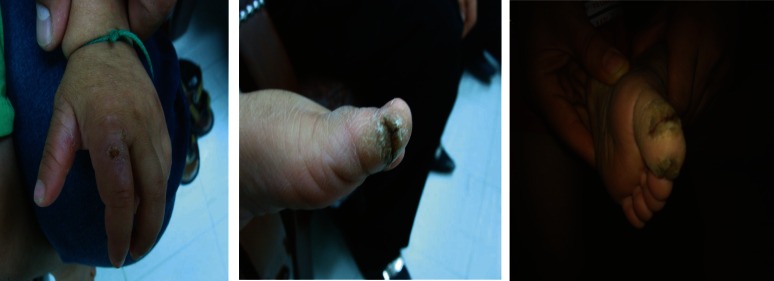
Ulcerative lesions of the fingers due to self-biting and palmar keratosis

A 2.5-year-old boy was referred to the pediatrics clinic with severe self-mutilating injuries to his hands, feet, tongue and oral mucosa caused by unconscious biting (Figure 1).

He is the second child of cousin parents and the product of a term, normal vaginal delivery with a birth weight of 3300 gr.

At two-months of age, he was admitted to the hospital as a result of fever and convulsion. The patient continued to have fever throughout his hospital stay and evaluation for the etiology of prolonged fever was done and abnormal results were not seen in any of the diagnostic tests including blood culture, CSF analysis and culture, urine analysis and culture, echocardiography, blood smear, bone marrow aspiration and culture, Wright and Vidal test, abdominal sonography and brain CT scan. Finally, he was discharged from the hospital on phenobarbital medication, but episodes of hyperthermia and unexplained fever recurred.

His parents found him never sweating after birth and he did not tolerate warm weather or sun exposure and he was irritable and cried with appropriate tearing in such situations. The parents also complained that the child showed no response to any kind of injury including pinpricking, burning, hitting and cutting. The boy had never complained of painful sensation and his hands or feet were burned by heater flame or hot water repeatedly. 

He had a right third metatarsal fracture without reason when he was 2.5 years old that was managed on an outpatient basis and caused edema and deformity in his right foot.

Teething had started at seven months of age, but because of biting and ulcerative lesions in the gums, his teeth had started to shed at 1.5 years of age.

On examination, he had 12 kilograms weight (10^th^ percentile), 92 centimeter height (75th percentile) and 48 centimeter head circumference (10th percentile) and a normal blood pressure without orthostatic hypotension. Ulcerative lesions were seen in his fingers, toes and mouth that were caused by self-biting (Figure 1).

**Figure 2.**
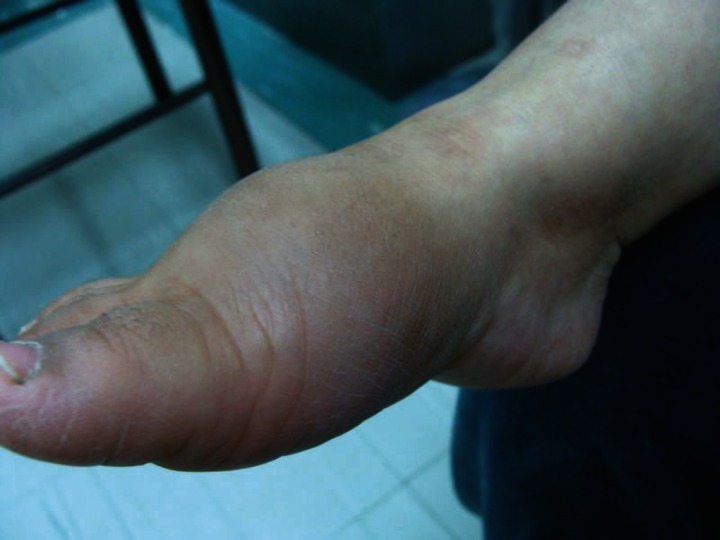
Charcot joint in the right ankle

Fungiform papillae on the tongue were present. In addition, keratosis and thick and dry skin in the palms of his hands and soles of feet were visible (Figure 1). He had red eyes and corneal ulcer. The deformity as Charcot joint (neuropathic osteoarthropathy) was observed in the right ankle due to repeated trauma (Figure 2).

Neurologic examination revealed normal function of the cranial nerves. The light response of pupils and deep tendon reflexes (DTR) were normal and plantar reflex was flexor bilaterally. He could not cooperate with the sensory exam. The child had neurodevelopmental delay based on Denver Developmental Screening Test-II. His parents and brother were healthy with normal intelligence. Family history was also negative and they had no sign of hereditary disease. The result of CBC, uric acid, serum glucose, liver, renal and thyroid function tests, serum lactate, ammonia, creatinine phosphokinase level and chromatography of amino acids were normal. Nerve conduction velocity (NCV) was normal and brain MRI showed mild brain atrophy. Electron microscopic study of nerve biopsy and genetic tests were not available in our center.

## Discussion

HSAN type I is the most common type of HSAN with autosomal dominant inheritance. The onset of symptoms occurs in the second to fourth decade of life with progressive sensory loss and chronic perforating ulcers of the feet and progressive destruction of the underlying bones. Sweating is impaired and NCV is normal.

HSAN type II is an autosomal recessive trait with the onset of symptoms in early infancy or childhood with loss of pain, temperature, pressure and light touch sensation and chronic ulcerations and multiple injuries to the fingers and feet. Deep tendon reflexes are reduced. 

Sensory nerve action potentials are reduced or absent.

HSAN type III or familial dysautonomia is an autosomal recessive disorder which occurs exclusively in Ashkenazi Jewish families. Nervous system dysfunction is usually evident from birth as a poor or absent suck reflex, hypotonia and hypothermia. Reduced or absent tears, absence of fungiform papillae, absent corneal reflexes, anhidrosis, decreased or absent DTR, postural hypotension, decreased response to pain and temperature perception, but normal touch perception become prominent subsequently. Many patients have cyclic vomiting and recurrent pneumonia. Intelligence remains normal. Many patients die from the disease during infancy or childhood.

HSAN type IV or congenital insensitivity to pain and anhidrosis is an autosomal recessive condition. The first signs are defects in thermoregulation and recurrent episodes of hyperthermia and unexplained fever that may be associated with seizures that begin in early infancy. Other symptoms often include anhidrosis (inability to sweat), profound loss of pain sensitivity, mild to moderate mental retardation. Microcephaly is often present. Insensitivity to pain and mental retardation may cause self-mutilation in these patients, especially in the fingers, lips and tongue. Corneal lacerations, non-painful fractures and Charcot arthropathies are commonly present. Joint deformities lead to chronic osteomyelitis and septic arthritis. The palmar skin is dry, thickened and hyperkeratotic. Children respond to tactile stimulation, and DTRs are often preserved. Autonomic deficits, including the inability to sweat in response to heat or chemical stimuli (e.g. pilocarpine) and the production of a wheal, but not a flare after intradermal histamine injection, are present, but touch and pressure sensitivity are unimpaired. In contrast to the other sensory neuropathies, motor and sensory nerve action potentials are normal.

HSAN type V is an autosomal recessive disorder phenotypically similar to HSAN IV presenting with loss of pain and temperature sensation (but other sensations preserved) and anhidrosis. Muscle strength, reflexes, and nerve conduction studies are normal. The main difference between type IV and V was thought to be the pattern of nerve fiber loss, and greater severity of anhidrosis in HSAN IV and lack of mental retardation in patients with HSAN V (1, 4, 5, 8). 

Based on the age of onset of symptoms, pattern of inheritance, clinical and paraclinical finding, our case seems to be consistent with HSAN type IV.

Schwarzkopf et al. found 37 different mutations at the NTRK1 in 28 known cases of CIPA in Israel (9). In another study in Israel, 18 percent of 33 cases of CIPA had osteomyelitis of the mandible (10).

Our case had corneal ulcer. Twenty seven percent (18/52) of reported CIPA patients until 2002 had corneal involvement (11). Since CIPA patients are asymptomatic even when they develop corneal ulcer as our case, it is logical to recommend visual and eye follow-up regularly in CIPA patients for early diagnosis and timely treatment of corneal neurotrophic ulcer and prevention of corneal and vision-threatening complications such as scarring and perforation (12).

Primary tooth loss and palmar hyperkeratosis were seen in our case as in the reported case by Bonkowsky et al. (13).

Osteoarticular and orthopedic complications of CIPA patients are one of the most common problems in such patients that need early recognition and timely treatment and also prevention of accidental injuries (4).

Early diagnosis and specific dental management in CIPA patients may be useful in reducing the frequency and severity and also preventing self-mutilation complications of this disorder (14).

Prenatal screening is the sole accessible option to prevent the birth of an affected child as no cure is available (9). 

**In conclusion**, in families who have a CIPA patient, prenatal screening should be done to prevent the birth of another affected child. Early recognition of CIPA patients and its orthopedic complications, prevention of accidental injuries, regular visual and eye followup and specific dental management could be useful in reducing the frequency and severity of complications in this disorder.
